# Isolation, Serotyping, and Molecular Detection of Bovine FMD Virus from Outbreak Cases in Abaʼala District of Afar Region, Ethiopia

**DOI:** 10.1155/2020/8847728

**Published:** 2020-12-09

**Authors:** Teshager Dubie, Tsedale Amare

**Affiliations:** ^1^Research and Community Service, Samara University, P.O. Box 132, Samara, Ethiopia; ^2^College of Veterinary Medicine, Samara University, P.O. Box 132, Samara, Ethiopia

## Abstract

**Background:**

On the basis of FMDV outbreak cases, a cross-sectional study was undertaken to collect samples from January 2019 to March 2020 intended for isolation, serotyping, and molecular detection of FMDV in the study district. The purposive sampling method was applied to select the study area for the reason of the presence of FMD outbreak case report during the study period. Totally, 27 FMD clinical samples were collected from affected study population during field outbreak. Out of 27 samples, 18 of them were inoculated on cultured Baby hamster kidney (BHK-21) monolayer cells, and all 27 samples were tested using conventional RT-PCR and sets of specific universal primers. Finally, the PCR products were visualized with UV illumination and imaged with gel documentation system.

**Results:**

The current study results revealed that out of 18 clinical samples subjected to virus isolation, 72.2% (*n* = 13) of these cultures exhibited FMDV-induced cytopathic effect (CPE) and the identified serotype was SAT-2 FMD virus. Out of 27 clinical samples tested by conventional RT-PCR, only 12 FMDV samples were found to be FMDV positive by universal primers. Out of 27 samples detected by conventional RT-PCR, only 12 FMDV samples were found to be FMDV positive by universal primers.

**Conclusions:**

Our study finding indicated that FMDV is prevalent in the study area and FMDV serotype SAT-2 was the causality for the outbreaks of the disease in the study area. Hence, region-wise FMD outbreak investigation, further phylogenetic analysis, and vaccine matching field isolates should be carried out for effective vaccine development to control the disease.

## 1. Background

Ethiopia owns the largest livestock population in Africa with an estimated animals' number of 59.5 million heads of cattle, 30.5 million sheep, 30.3 million goats, and 54.5 million chickens [1]. The role of livestock resource to the nation's economy is expected at 19% from the total GDP, 45–48% of the agricultural GDP and about 20% of the country's export incomes [2]. Moreover, the livestock sector plays a vital role in the livelihood of popular of human population in Ethiopia as a source of meat, milk, drought power, and income. The contribution of livestock to the country's economy primarily in terms of foreign currency gains is via export of live animals, meat, and skin and hides [3]. In spite of possessing such huge amount of livestock resources, the country could not utilize the sector as a total as a result of extremely rampant livestock diseases, absence of appropriate disease management strategy and less attention from government side [4, 5].

Livestock diseases remain the most vital impediments to the development of the sector through reducing production and productivity that could ultimately affect regional, national, and international trade in live animal and animal products' [4]. According to [6], livestock diseases cause major economic losses to the peasant farmer and pastoralists in Ethiopia amounting to hundreds of millions of birr every year. Approximately, annual mortality rates attributed to these livestock diseases is computed to be 9–12% for cattle herds, and 15% and 13% for sheep and goat flocks, respectively [7]. Among livestock diseases hindering production and productivity of the sector, foot and mouth disease (FMD) is the most known economically important transboundary viral disease of cattle in Ethiopia [6, 8]. Foot and mouth disease (FMD) is an extremely contagious and extremely infectious livestock disease of all cloven hoofed animals. It is the world's most important cattle disease and accountable for vast worldwide drop of livestock production and encouraging national and global business impediments for livestock and livestock products [4, 9]. Foot and mouth disease (FMD) is caused by FMD virus (FMDV) that comes under the genus *Aphthovirus* within the family Picornaviridae. Clinically, FMD is manifested by fever, loss of appetite, salivation, vesicular eruptions in mucosa of the mouth, skin of the interdigital spaces and coronary bands of the feet and teats, and sudden death of young stock [10, 11]. As of the International Organization for Animal Health (OIE), FMD ranks first among globally important notifiable infectious livestock diseases because of exports of infected livestock and livestock products could easily cause outbreak in countries that are previously free from FMD outbreak cases and transboundary distribution nature of the disease [12]. Pastoralists are highly impacted by direct and indirect effects of FMD as their lives are straightforwardly dependent on livestock production [6, 13]. Generally, studies conducted on FMD serostatus previously indicated the presence of the disease in various areas of the country with seroprevalence that ranges from 8.18% to 44.2% in different part of the nation [14, 15].

Foot and mouth disease virus (FMDV) has seven immunologically and genetically distinct serotypes (O, A, C, Asia 1, Southern African Territories (SAT)-1, SAT-2, and SAT-3) that cause indistinguishable clinical disease [16]. Within these serotypes, over 65 diversities of topotypes, genetic lineages, and strains have also been identified using biochemical and immunological tests. Currently, five FMDV serotypes (O, A, C, SAT-1, and SAT-2) are identified and documented in Ethiopia [3, 4, 17, 18]. The serotypes also differ in their geographical distribution over the world as well as in many regions of the country [3, 19]. According to [20] retrospective study finding, FMDV serotypes O, A, SAT 2, and SAT 1 were identified as the causative serotypes for outbreak cases occurring during the study time 2007–2012. While O was the dominant serotype, SAT 2 was the serotype which indicated raise in comparative frequency of occurrence [3, 20]. Prompted investigation and detection of FMDV serotypes during outbreak was highly crucial to determine the origin of infection and to use appropriate vaccine [21].

Despite occurrence of several outbreaks of FMDV in the Afar region, there is no even single documented information to know about the disease current serostatus, serotypes circulating in the region in general, and the study area in particular. To develop effective control measures of FMD, determining its serostatus, virus isolation, and identification of the serotype(s) circulating in a particular area would have paramount importance. Moreover, having a detailed knowledge on the specific serotypes circulating in a particular area has paramount importance for companies to target for each specific FMDV serotype for effective vaccine development in steady of rely on production of trivalent vaccine for serotype O, A, and SAT-2.Therefore, the present study was intended for isolation and serotype identification of FMDV from outbreak cases in Aba'ala District of Afar Region from January 2019 to March 2020 in Ethiopia.

## 2. Materials and Methods

### 2.1. Description of the Study Areas

This research work was implemented in Aba'ala District (Erkudi and Hidmo kebeles), which is located in Afar Region, Ethiopia. This study district was purposively selected for the reason that the presence of active FMD outbreak case report in the course of the study period, January 2019 to March 2020. Afar Regional State shares joint intercontinental borders with Eritrea in the northeast and Djibouti in the east part of the region. The region is described specifically through arid and semiarid weather conditions with low and unpredictable rainfall. The altitude of the region ranges from 120 m below sea level in Danakil depression to 1500 m above sea level. Majority of the pastoral community mainly depend on livestock production for their livelihood. According to APADB (2006), approximately there are 1.9 million cattle population in the region, and 90% of the study animals are managed under pastoral production and the rest 10% in the agropastoral production system. The study area is situated in the north area of the region, northeastern Ethiopia. It lies around between 13°15′ and 13°30′ 1atitude and 39°39′ and 39°55′ longitude. The high temperature of Afar ranges as of 25°C in case of the rainy period to 48°C during the dry season [22].

### 2.2. Study Population

The study populations were cattle that had experienced outbreak cases of FMDV and manifested typical FMD clinical signs in the Abaʼala district area during the study period of this research work. The study animals were cautiously inspected for the manifestation of distinguishing clinical signs of FMD such as vesicular lesions around the oral cavity, on the feet, salivation, lameness, anorexia, and rise in temperature [23]. All ages and sexes of the study population reared by agropastoralists in the outbreak-affected kebeles (subunits) of the study district were sampled.

### 2.3. Study Design

Prior to field-level investigation and sample collection, district- and kebele-level animal health expertises were informed to report for regional veterinary laboratory centers when FMD outbreak occurred. Therefore, based on the occurrence of active FMD outbreak case report and active outbreak finding, a cross-sectional study was used to collect tissue samples. Clinically, FMD-suspected study populations were physically inspected for the manifestation of FMD with typical signs were sampled to collect biopsy samples that were intended for viral isolation, molecular detection, and serotype identification purpose.

### 2.4. Sampling Techniques and Sample Size Determination

**Figure 1:**
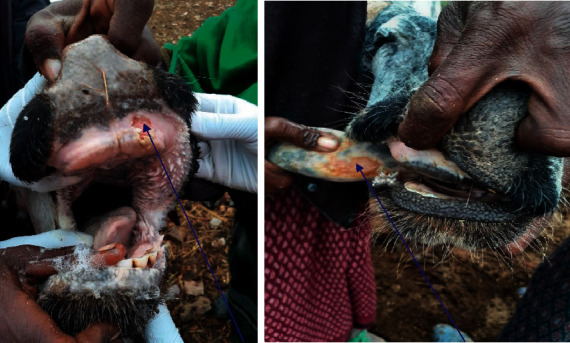
Gum and tongue lesion observed on cattle infected with foot and mouth disease.

The purposive sampling method was employed to select FMD-affected study district, cattle herds, and sampling animals as a result of the occurrence of FMD active case reports in the course of the study period, January 2019 to March 2020. Accordingly, within the study areas (subunits), animals with clear signs and symptoms and animals which are suspected to be infected with FMDV as indicated in Figure 1 were selected and sampled. From all outbreak-affected kebeles, 27 swab, epithelial tissue and vesicular fluid samples were collected from clinically FMD-suspected animals with active outbreak lesions for cell culture-based virus isolation, molecular detection, and identification of serotypes circulating in the study district.

### 2.5. Sample Collection and Transportation

A total of 27 representative samples (6 epithelial tissue samples, 5 vesicular fluid samples, and 16 swab samples) were collected with the help of tissue forceps from unruptured and freshly ruptured vesicles of clinically affected animals during the course of field outbreak to isolate the circulating viruses responsible for the occurrence of disease. These collected FMD-suspected samples were kept in a sampling bottle containing a virus transport medium that has equal volume of 0.04 M phosphate buffer saline (PBS) with 50% glycerol enriched by antibiotics and antifungal according to the protocol recommended by OIE [18]. Collected clinical FMDV-suspected specimens were transported to laboratory and stored at −20°C and got transportation to the National Veterinary Institute (NVI) using cold chain for virus isolation, molecular detection, and serotype identification purpose.

### 2.6. FMD Virus Isolation

The samples collected were processed and cultured on bovine hamster kidney (BHK-21) cell monolayer with three subsequent passages as follows. About 1 gram of each tissue was taken and washed three times using sterile phosphate buffered saline containing antibiotics and antifungal (PBS) on Petridish. Then, washed tissues were transferred to sterile mortar, cut into pieces using scissor and minced by scalpel blade. These minced tissues were then grounded and homogenized in sterile sand with a sterile pestle and mortal. Nine ml of PBS was added to the homogenized tissues and well mixed, and small volume tissue culture made and small amount of five percent antibiotics (penicillin, streptomycin, and Amphotericin B solution) containing the medium were added so that the final volume was ten times that of the epithelial tissue, producing of ten percent suspension [18]. All procedures were conducted under the Biosafety cabinet level 2. About 1 ml of filtered tissue suspension was inoculated on confluent cultured Baby hamster kidney (BHK-21) monolayer cells grown on 25 cm^2^ tissue culture flasks and incubated at 37°C for 1 hr for adsorption of the virus. Then, cell cultures were added 8 ml of the maintenance medium (2% MEM) and incubated at 37°C and 5% CO_2_ in a humidified incubator. The appearance of virus-induced cytopathic effect (CPE) was observed daily under the inverted microscope. The inoculated cell line was harvested when 85–100% of CPE was observed. These infected cells did not show CPE within 72-hour postinfection on the third passage were supposed to be virus negative [18, 24]. Samples that showed typical CPE (positive cases), clinical tissue materials were used for serotype identification of the virus involved in the outbreak cases using antigen detection sandwich ELISA [25].

### 2.7. Serotyping of FMD Virus Isolates

FMD serotyping was executed using both antigen detection sandwich ELISA and sets of serotype specific primers intended for testing of FMD virus and identifying the serotypes responsible for outbreaks cases. Sandwich‐ELISA was executed with particular combinations of anti‐FMDV monoclonal antibodies (MAb), used as coated and conjugated antibodies. The kit was developed for detection and serotyping of FMDV O, A, C, SAT-1, and SAT-2. A pan‐FMDV test, detecting any isolates of O, A, C, Asia1, and SAT serotypes, was also included in the kit to complement the specific serotyping of FMDV. The test was implemented based on the manufacturer's instruction and OIE [26]. A total of 13 positive sample suspensions that exhibited FMDV cytopathic effect (CPE) on BHK-21 cell were needed to be tested for detection of serotype identification using sandwich ELISA on a microplate containing 96 wells.

About 25 *μ*l of dilute buffer was dispensed into all wells of the test plate, and then 25 *μ*l of previously diluted samples using ELISA buffer and ready‐to‐use controls was dispensed into the appropriate wells of the test plate precoated with recombinant FMD viral antibody. One positive control for each FMD types O, A, SAT-1, and SAT-2 and negative controls were included in each plate. The plates were sealed using the enclosed plate sealer and incubated for 1 hr at room temperature (20–25°C). After incubation, all fluids on the plates were discarded and the remaining residual fluids were removed. Then, 200 *μ*l of washing solution was added and incubated for 3 min at room temperature; subsequently, wells were emptied and the washing was repeated twice (three washing cycles in total). Then, all residual fluids were removed by tapping on clean absorbent paper and 50 *μ*l of conjugate. A was added from columns 1 to 8, and the same volume of conjugate B was added from columns 9 to 12. Plates were covered and incubated at room temperature for 1 hour. After incubation, 50 *μ*l of substrate per well was added to all wells and plates were covered and left at room temperature for 20 minutes in the dark. The reaction was stopped by adding 50 *μ*l of stop solution (sulfuric acid (H_2_SO_4_)). Immediately after stopping, reading the optical density (OD) of each well was done at 450 nm wavelength using microplate reader.

### 2.8. Molecular Detection of FMD Virus

The presence of FMD viral genetic material in all 27 collected field samples was tested using conventional RT-PCR and specific primers that amplify viral protein 1 (VP1) of FMDV using the RNeasy Mini Kit following the manufacturer's instruction (Qiagen, USA).

### 2.9. FMD Viral RNA Extraction and PCR Amplification

Total RNA was extracted from collected FMD-suspected clinical sample suspension using Qiagen RNA extraction kit following the manufacturer's instructions as [27]. Briefly, 140 mircoliters of sample suspension was added to 560 *μ*l buffer AVL carrier RNA in the mircocentrifuge. The tubes were briefly centrifuged to remove drops from the inside of the lid. Then, 560 *μ*l of ethanol (70%) was added to the sample and mixed by pulse vortexing for 15 seconds followed by centrifuging to remove drops from the inside lid. Then, 630 *μ*l of the solution was applied to the QIAMP Mini-spin column in a 2 ml collection tube and centrifuged 12,500 rpm for 1 min. The filtrate was discarded, and the column was placed in a fresh 2 ml collection tube. Then, 500 *μ*l of buffer AW2 were added and centrifuged at 12,500 rpm for three min and the filtrate was discarded. Next, 65 *μ*l of Buffer AVE was added to the column equilibrated at room temperature for one min and centrifuged at 12,500 for 1 min. Using reverse transcription polymerase chain reaction (RT-PCR) and specific primers set FMDV7-forward (FMDV7F) and FMDV7-reverse (FMDV7R) as depicted in Table 1, extracted RNA samples were detected for the presence of FMDV.

### 2.10. Agarose Gel Electrophoresis

The PCR products were analyzed on the prepared 1.5% Agarose gel by adding 4 *μ*l gel red with loading dye, then the PCR product was loaded in the volume of 10 *μ*l in each well, and 10 *μ*l molecular marker (ladder) was added started 100 bp plus. Electrophoresis was run for one hour at 120 V. Then, the DNA band was visualized by UV illumination, using desktop according to the base pair (bp), and then the size was determined and documented.

### 2.11. Data Management and Statistical Analysis

Data generated from laboratory investigations were recorded and coded using Microsoft Excel spreadsheet and analyzed using STATA version 14.2 for Windows. Cell-culture results, CPE development, and molecular characterization results were recorded and tabulated. Virus isolation and molecular detection of FMD virus were elaborated using descriptive statistics analysis. Moreover, regarding the molecular characterization, the banding patterns of individual sample were scored based on the presence or absence of the bands with the appropriate base pairs.

## 3. Results

### 3.1. Clinical Examination of FMD Outbreaks

In this outbreak investigation and sample collection, characteristic clinical signs of FMDV in the study population were salivation, lameness, vesicle formations on oral cavity, and interdigital vesicles. Suggestive lesions of FMD on the mouth contained destructions and sores on the upper and lower pad area and tongue, while feet abrasions consist of wearing away on the interdigital space. These study populations were reluctant to travel and lagging behind the healthy cattle and deny for feeding.

### 3.2. FMD Virus Isolation

**Figure 2:**
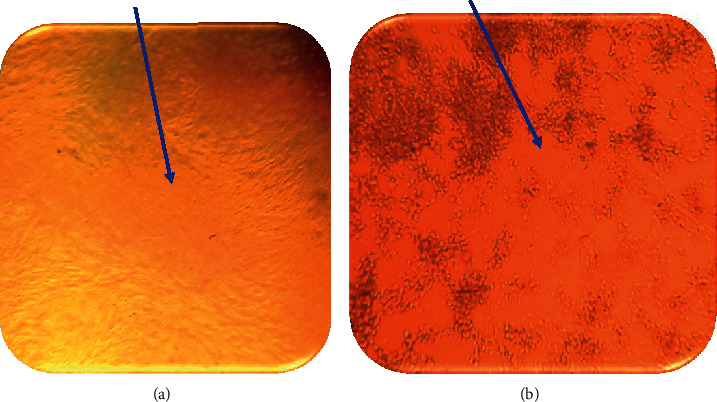
Picture taken during FMDV virus isolation: (a) BHK-21 cell control (cell without FMDV infection); (b) virus-induced cytopathic effect (indicated by arrow) inoculated with FMDV outbreak isolates.

The current cell culture-based FMD virus isolation result revealed that out of 18 suspected clinical samples processed and cultured, 72.2% (*n* = 13) representative samples exhibited morphological alterations (FMDV cytopathic effect (CPE) on BHK-21 cells, while the other clinical samples of FMDV (*n* = 9) did not inoculated on the cells because these samples were collected from the same outbreak in the study areas. Out of 13 FMD clinical samples that showed cytopathic effect on BHK-21 cells, 33.3% (*n* = 6), 22.2% (*n* = 4), and 16.7% (*n* = 3) were epithelial tissues, vesicular fluid, and swab samples, respectively. These FMD positive clinical samples were characterized primarily by a quick sloughing of BHK-21 monolayer cells and these sloughed cells were roughly round, swelling, and formed singly in shape (Figure 2). As time progress, there was sloughing of cells or monolayer detachment from the wall of the cell-culture flask and even some cells were severely damaged within 72 hrs after inoculation and finally cell death that indicates the presence of virus. However, samples that did not show CPE do not induce morphologic changes of cell. Whole cell sloughing of the pane was regularly observed after 48 hrs of cell injection. Figure 2 illustrates the circular and lysis (CPE) of BHK-21 cells injected by the virus and uninfected ones.

### 3.3. Serotyping of FMD Virus Isolates

FMD serotyping was executed using 13 positive samples by antigen detection sandwich ELISA intended for identifying the serotypes involved in the outbreaks. Out of 13 positive FMDV clinical samples, all FMDV positive samples were identified to be serotype SAT-2. Therefore, the current study finding revealed that SAT-2 serotype could be the possible causes of the disease detected in the outbreak areas as depicted in Table 2. All outbreaks confirmed samples collected from both kebeles of the same district were identified as SAT-2 serotype. In conclusion, outbreaks of the study district (subunits) were occurred due to FMD serotype SAT-2.

### 3.4. Molecular Detection of FMD Virus

The extracted RNA from all 27 FMD suspected clinical samples was detected using the conventional RT-PCR method and specific primers [28]. This conventional RT-PCR was employed for the amplification and detects the genetic material of the disease in collected clinical samples [29]. All samples were amplified and detected using FMDV universal primers (FMDV7F/FMDV7R). Out of 27 samples detected, only 12 FMDV clinical samples were found to be FMDV positive (DNA bands on gel electrophoresis around 328 bp).

## 4. Discussion

The present study was the first in its kind about foot and mouth disease isolation, molecular detection, and identifying of the serotype involving in Afar Region. Foot and mouth disease (FMD) is responsible for frequent outbreaks and causes significant economic devastation in the region in particular and on the nation in general. The disease is described by development of typical FMD lesions around the mouth as well as on the foot and unexpected losses of newborn calves [10, 11]. Occurrence of the disease epidemics is growing livestock problems entirely in all corners of the country. The disease has become one of the most important bottlenecks to livestock keepers as result of significant reduction in production and productivity as well as possibly trade restriction in Afar Region in particular and Ethiopia in general [30–32].

In this research finding, from 18 suspected clinical samples subjected to BHK-21 cell line adaptation, 72.2% (*n* = 13) field samples showed FMDV-induced cytopathic effect (CPE). These cells were appeared as rounding in cell culture, swelling, and clumping of the cells as one can demonstrate from Figure 1. The present study finding was consistent with previous research works in which positive sample (CPE) on BHK-21 cells was described by a fast sloughing of the cells [33–35]. Our study result was in line with study finding by [36], in which infected cells in both study results showed round and sloughing as well as monolayer detachment from the wall of the cell-culture flask. Other authors also described that FMDV isolated from clinical samples and inoculated on BHK-21 cell-culture results in infected cells that showed specific CPE within 24–48-hour postinfection was characterized by rounding of cells and distortion of the monolayer and cell detachments [37]. The remaining samples did not show CPE; this could be due to loss of our samples through shipping from sample collection site to laboratory analysis.

Ethiopia is one of the FMD endemic countries in the horn of Africa, with almost five serotypes prevailing so far. Cumulative research reports in Ethiopia on FMDV serotypes revealed that this disease occurrence is due to any of O, A, C, SAT-2, and SAT-1 as diagnosed by clinically, serologically, and molecular techniques during the period 1981–2018 [3, 20]. In our study result, serotyping of FMDV results disclosed that the identified serotype SAT-2 (100%) FMD virus was circulating in Aba'ala District of Afar Region. This could be to mean that serotype SAT-2 is vastly prevailing and the foremost serotype is responsible for frequent outbreaks in the study area of Afar Region, Ethiopia. In support of this study findings, studies conducted by other authors [3, 13, 32, 38] showed serotype SAT-2 virus in Borean pastoral area, Benishangul-Gumuz, Gambella, Addis Ababa, and Adama, respectively. Moreover, serotype SAT-2 was previously reported from many Sub-Saharan African countries [39, 40] described the endemicity of this serotype in these countries. Studies conducted in Uganda indicated that SAT-2 serotype was the most prevalent serotype accountable for the disease occurrence [31]. Another FMDV serotyping study results in Chad in 2016 showed that SAT-2 was the dominant serotype during its study period followed by serotype O [41]. Furthermore, the International Organization for Animal Health (OIE) FMD disease occurrence report in Africa continent since 2000–2010 disclosed that SAT-2 was escalating as an important serotype (41%) followed by O serotype (23%) [42]. Multi-topotype SAT-2 endemicity and outbreaks out of the Sub-Saharan terrestrial ranges have also been observed in countries south of the Sahara desert, and the Northern African and the Middle East region such as Libya, Egypt, Palestinian Autonomous Territories (PAT), and Bahrain [43].

In this study, out of 27 clinical samples detected using conventional PCR for the presence of FMDV genetic material in the sample, only 44.4% (*n* = 12) were found to be positive. Of these 12 samples detected as positive for FMDV, bovine epithelial tissues were accounted for 22.2% (*n* = 6) and had the lower Ct values which could indicate higher concentrations of the virus in these samples. Our results also showed that bovine vesicular fluid samples were accounted for 14.5% (*n* = 4) and swab samples were accounted for 7.4% (*n* = 2). This study finding confirmed the existence of more FMD viral RNA in the epithelial tissue samples as compared to vesicular and swab samples. This study finding is supported by OIE [18] as this institution described epithelial tissues are the ideal samples for virus detection. The presence of SAT-2 serotype in the present study district would be as result of uncontrolled cross‐border movement of animals intended for pursuit of feed and water and also free trade in livestock among neighboring regions and countries since SAT-2 is widespread to various neighboring countries [44–46].

## 5. Conclusions

The present study finding indicated that FMDV is prevalent in the study area of Afar Region as confirmed by clinically, serologically, and molecular techniques particularly in the study area of the region and serotype SAT-2 was the causality for the outbreaks of the disease in the study area. The occurrence of this disease is a foremost badly behaved for the improvement of the livestock industry as it causes enormous worldwide harms of livestock sector as well as severe impacts on export earing from national and international trade thereby threaten the living means of livestock keepers in particular and income source of the country in general. Out of the serotypes identified in our country, the identified prevailing serotype was SAT-2 that causes frequent outbreaks in the study area of Afar Region, Ethiopia. Region-wise regular FMD outbreak investigation to have more full information about the serotypes, topotypes involving in the region, and vaccine matching studies of field isolates to evaluate vaccine protection potential has paramount important for effective vaccine development.

## Figures and Tables

**Table 1:** The Universal primers and thermal cycle used for amplification of FMDV.

Primer-FMDV7 universal-forward 5pm/*μ*l 5ʹ-GCCTGGTCTTTCCAGGTCT-3ʹ			
Primer-FMDV7 universal-reverse-5pm/*μ*l 5ʹ-CCAGTCCCCTTCTCAGATC-3ʹ			
	Temperature	Time	Cycle
Initial denaturation	95	5 min	1 cycle
1^st^ denaturation	94	1 min	35 cycles
Annealing	54	1 min	
Elongation	72	1 min	
Final elongation	72	10 min	1 cycle

**Table 2:** Types of samples and serotyping results of FMD virus isolates at NVI Ethiopia.

No	Sample code	Sample type	Sex	Kebele-level outbreaks	Species	Serotyping results
1	E-M-E1	Epithelial tissue	Male	Erkudi	Bovine	SAT-2
2	E-M-V2	Vesicular sample	Male	Erkudi	Bovine	SAT-2
3	E-F-S3	Swab sample	Female	Erkudi	Bovine	SAT-2
4	E-M-E4	Epithelial tissue	Male	Erkudi	Bovine	SAT-2
5	E-F-V5	Vesicular sample	Female	Erkudi	Bovine	SAT-2
6	E-F-S6	Swab sample	Female	Erkudi	Bovine	SAT-2
7	E-F-E7	Epithelial tissue	Female	Erkudi	Bovine	SAT-2
8	E-F-V8	Vesicular sample	Female	Erkudi	Bovine	SAT-2
9	E-F-E9	Epithelial tissue	Female	Erkudi	Bovine	SAT-2
10	E-F-V10	Vesicular sample	Female	Erkudi	Bovine	SAT-2
11	E-M-E11	Epithelial tissue	Male	Erkudi	Bovine	SAT-2
12	E-F-S12	Swab sample	Female	Erkudi	Bovine	SAT-2
13	E-F-E13	Epithelial tissue	Female	Erkudi	Bovine	SAT-2

Key: *E* = Erkudi; H = Hidmo; M = male; F = female; S = swab; V = vesicular; SAT-2 = Southern African Territories; En = epithelial tissue where, *n* = 1, 2,…, n.

## Data Availability

The data sets used and analyzed during the current study are available from the corresponding author on reasonable request.

## Abbreviations

CSA: Central Statistical Authority
FAO: Food and Agriculture Organization
FMD: Foot and mouth disease
FMDV: Foot and mouth disease virus
GDP: Growth domestic product
NVI: National Veterinary Institute
OIE: International Organization for Animal Health
SAT: Southern African Territories
TD: Teshager Dubie
TA: Tsedale Amare.
